# Classification and Anti-*Streptococcus mutans* Mechanism Summary of Chinese Botanical Products

**DOI:** 10.3390/pathogens15030280

**Published:** 2026-03-04

**Authors:** Yuelin Li, Zhongyi Fang, Ruijie Huang

**Affiliations:** State Key Laboratory of Oral Diseases, National Center for Stomatology, National Clinical Research Center for Oral Diseases, Department of Pediatric Dentistry, West China Hospital of Stomatology, Sichuan University, Chengdu 610041, China; qingmu2214@163.com (Y.L.); 19940712261@163.com (Z.F.)

**Keywords:** Chinese botanical products, traditional Chinese medicine, dental caries, *Streptococcus mutans*, caries prevention

## Abstract

Dental caries, one of the most prevalent diseases worldwide, poses a significant threat to oral health. *Streptococcus mutans* is one of the key pathogenic bacteria associated with dental caries. Numerous Chinese botanical products (CBPs) have been shown to possess antibacterial effects against *S. mutans*. However, given the wide variety of CBPs that have been investigated, a systematic summary of their effects is needed. To address this need, in the present review, CBPs are categorized into five groups based on their major bioactive components: organic acid-based CBPs, alkaloid-based CBPs, phenol-based CBPs, anthraquinone-based CBPs, and other types. In addition to their chemical composition, the conventional use, pharmacological effects, and toxicity of these CBPs are also discussed, followed by an exploration of their anti-*S. mutans* mechanisms, including the synthesis of biofilm scaffolds and water-insoluble glucans, energy metabolism and soluble glucan production, acid generation and tolerance, bacterial cell integrity, remineralization processes, and intercellular communication via quorum sensing (QS). In summary, it is suggested that CBPs have considerable benefits in caries prevention and could be promisingly applied in clinical treatments.

## 1. Introduction

Dental caries, a chronic progressive disease occurring on the hard tissues of teeth, is driven by multiple factors including microorganisms, oral environment, host and time [1]. It is one of the most prevalent diseases worldwide and has become one of the main causes of tooth loss over the years. In 2021, there were approximately 2.3 billion people suffering from permanent dental caries and 530 million children suffering from primary dental caries around the world [2].

*Streptococcus mutans* is one of the key pathogenic bacteria associated with dental caries due to its acidogenicity and aciduricity [3]. *S. mutans* utilizes sucrose to generate glucose and fructose, and further ferments to lactic acid or synthesizes extracellular polysaccharides (EPS) through glucosyltransferases (Gtfs) regulation. EPS is the main component of the cariogenic biofilm matrix [1].

CBPs have been used as folk medicines for thousands of years, while modern medicine tries to explore their bioactive components and identify their antibacterial effects. As early as the Western Han Dynasty (206 BC–AD 8), ancient Chinese physicians recommended rinsing the mouth with a decoction of *Sophora flavescens* to relieve tooth decay and oral discomfort, often in combination with acupuncture and moxibustion [4]. In recent years, with advances in modern medicine and molecular microbiology, many researchers have conducted extensive studies on CBPs and *S. mutans*. However, due to the complex and diverse nature of CBPs, a clear classification system is lacking, making it difficult to comprehensively understand their effects on *S. mutans*. This review aims to classify anti-*S. mutans* CBPs based on their main bioactive components and to further elucidate working mechanisms at the molecular level.

## 2. Classifications of Anti-*S. mutans* CBPs

**Figure 1:**
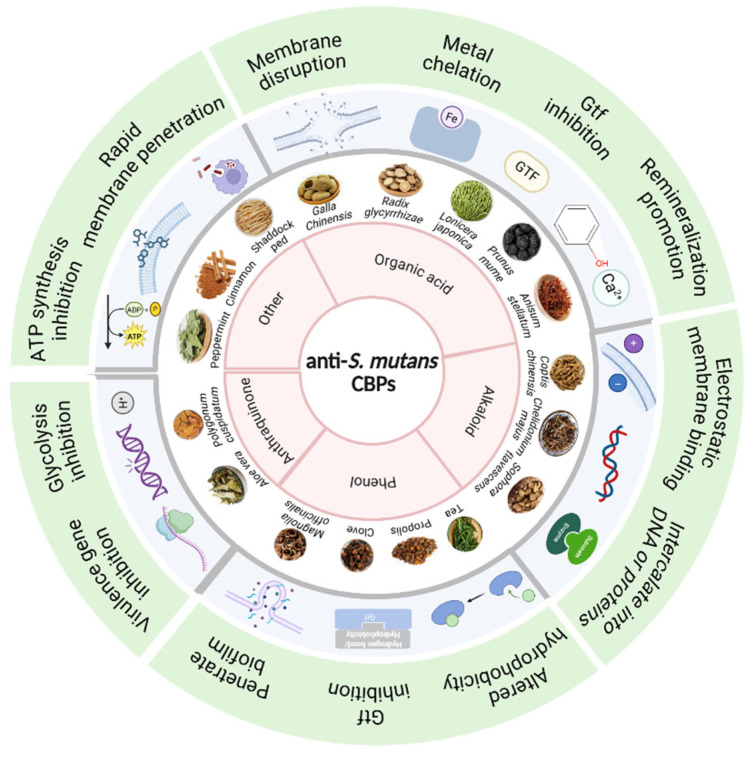
Schematic classification of anti-*S. mutans* CBPs based on major bioactive components. Created in BioRender. Li, Y. (2026) https://BioRender.com/1o3exy9 (Accessed on 25 February 2026).

Based on the properties and characteristics of bioactive components, CBPs are classified into five groups, organic acid-based CBPs, alkaloid-based CBPs, phenol-based CBPs, anthraquinone-based CBPs, and other types. The overall classification framework is visually demonstrated in Figure 1. Their classifications, names, bioactive ingredients, chemical structures, and sources are summarized in Table 1.

### 2.1. Organic Acid-Based CBPs

Organic acid-based CBPs include Galla chinensis (GC), licorice root (*Radix glycyrrhizae*), honey-suckle (*Lonicera japonica*), plum (*Prunus mume*), *Anisum stellatim*, etc. The organic acids are defined as acidic organic compounds, which are widely distributed in the leaves, roots, and fruits of CBPs.

The anti-caries effect of acid-based TCMs is rooted in their acidic bioactive ingredient. Organic acid, with its fat-soluble property, can diffuse across bacterial cell membranes to reach the interior of the cell to disrupt cell function [5]. Groups like carboxyl and hydroxyl can act as chelating agents and deprive bacteria of essential trace elements such as iron to interfere with their metabolic enzyme activity [6]. Specifically, some phenolic hydroxyl groups can simultaneously bind to the organic matrix and calcium ions in teeth, acting as a “molecular bridge”, enabling them to promote remineralization in a targeted manner [7].

GC is among the most frequently investigated anti-*S. mutans* CBPs, with tannic acid compound as the main effective component [8]. It was identified as a natural medical product as early as the Tang Dynasty (618-907 AD) in ancient China and was traditionally used for cough, bleeding, diarrhea, vomiting, sweating, and hemorrhoids [9]. Recent studies have revealed its antibacterial, antidiabetic, anti-inflammatory, and even antitumor effects [10]. GC yields over 50 constituents, with gallic acid (GA), methyl gallate (MG), and polymeric polyphenols exhibiting the highest efficacy in inhibiting caries [11]. Chemical structural analysis shows that GC mainly consists of gallotannins, which are composed of a glucose core surrounded by several tannic acid units [12]. Notably, different extracts isolated from GC express varying degrees of inhibitory ability. Among them, gallotannins, the aqueous extract of GC, showed the strongest antibacterial activity, followed by polyphenols [13]. Kang et al. [14] reported a stronger antibiofilm activity of MG than GA and they surprisingly detected that polyphenols additionally inhibit the activity of Gtfs.

*Radix glycyrrhizae* (licorice root) is another promising acid-based anti-*S. mutans* CBP. It is derived from the dried roots and rhizomes of *Glycyrrhiza uralensis* Fisch, *Glycyrrhiza inflata*, or *Glycyrrhiza glabra* [15]. The roots and rhizomes are harvested, dried, and processed into licorice root extracts, which are extensively applied in cosmetics, foods, and medicine. In medical applications, it plays an essential role in many pharmaceutical formulas and was originally applied in gastrointestinal diseases such as gastritis and peptic ulcers [16]. Research studies within recent decades have validated that licorice root extracts, especially the glycyrrhizic acid fraction, have a surface coating effect that protects the tooth surface from microbial attachment and suppress the activity of Gtfs at the same time [17]. Lollipops with licorice root extracts have been demonstrated to have good acceptance among children because of their mild sweet taste [18], which represents its possible application in early childhood caries management.

*Lonicera japonica* (honeysuckle), *Prunus mume* (plum) and *Anisum stellatum* are less studied but also found to prevent caries to some extent. Honeysuckle, the dried flower buds or flowers in the early blooming stage of *Lonicera japonica*, is known as Jin Yin Hua in traditional Chinese medicine [19]. The main pharmacological bioactive components of honeysuckle include chlorogenic acid and luteolin glycosides. Metabolomics and transcriptomics analyses revealed that chlorogenic acid could decrease dental plaque formation by downregulating the activity of Gtfs through the quorum sensing (QS) system and a transcriptional regulator [20].

*Prunus mume*, originating from the south of mainland China (named méi), has been used for a long time in Eastern Asia [21]. While it has been demonstrated that plum could lower the vitality of oral bacteria [22], patients might have difficulty in accepting the sour taste. *Anisum stellatum* is a culinary spice with medicinal value. It is the dried ripe fruit of *Illicium verum*, with shikimic acid as the main bioactive constituent [23]. Traditionally, shikimic acid inhibits platelet aggregation and thrombosis by affecting arachidonic acid metabolism and has analgesic and anti-inflammatory properties [24]. Simultaneously, Zhang et al. [25] pointed out that shikimic acid could also prevent caries by reducing plaque biofilm and virulence factors.

### 2.2. Alkaloid-Based CBPs

Alkaloids are nitrogen-containing compounds that are naturally occurring and have a variety of biological activities, including antimicrobial properties [26]. Alkaloid-based CBPs could exert an anti-caries effect through adsorbing onto the surface of the negatively charged bacterial cell membrane by electrostatic action, resulting in bacterial lysis and the release of cell contents [27]. The complex circular structures of alkaloids may also help them to embed into DNA or proteins, interfere with gene expression, and regulate enzymatic activity, thus effectively inhibiting cariogenic biofilm formation and maturation [27,28].

Berberine is a kind of isoquinoline alkaloid extracted from the rhizomes of *Coptis chinensis*, possessing a wide range of therapeutic values with few side effects for long-term use [29]. As a broad-spectrum antibacterial agent, especially against *Shigella dysenteria*, *Vibrio cholera* and *Salmonella typhi*, it has been continuously used to treat bacterial diarrhea since ancient times, including functional diarrhea or diarrhea-type irritable bowel syndrome [30]. Recent studies have reported a new discovery that berberine is promising for the prevention and management of caries, primarily through its inhibition of biofilm formation and suppression of cariogenic virulence factor expression [28].

*Chelidonium majus* (Papaveraceae) extracts exhibit antimicrobial activity due to their complex alkaloid composition [31]. At present, there were 94 alkaloids isolated and distinguished from *Chelidonium majus*, and the bioactive ingredients aporphines belong to the isoquinoline alkaloids [32]. Isoquinoline alkaloids are a class of alkaloids derived from phenylalanine or tyrosine, which have a wide range of pharmacological values, including antibacterial, antifungal, anti-inflammatory, antitumor, and antitussive effects [33]. Some scholars have further reported a significant inhibitory effect against *S. mutans* of chelerythrine, an alkaloid extracted from *Chelidonium majus*, primarily by reducing the adhesion ability of oral bacteria [34].

*Sophora flavescens*, also known as “Kushen”, is a deciduous shrub widely distributed throughout East Asia and commonly used for clearing heat, killing worms, and as a diuretic [35]. *Sophora flavescens* produces a wide range of secondary metabolites including multiple alkaloids. Among them, matrine and oxymatrine are the main bioactive ingredients, exerting anti-inflammatory, antioxidative, and immunosuppression effects [36]. It was reported that sophoraflavanone G isolated from *Sophora flavescens* was able to inhibit the growth of *S. mutans* around 0.5~4 μg/mL [37], and *S. mutans* was susceptible to *Sophora flavescens’s* other extract [38].

### 2.3. Phenol-Based CBPs

Phenolic compounds refer to hydroxyl derivatives of aromatic hydrocarbons, which display antibacterial effects against a variety of oral bacteria including *S. mutans* [39]. Due to the polyphenol chemical structure, phenol-based CBPs can usually strongly bind proteins and polysaccharides through hydrogen bonding and hydrophobicity with phenol hydroxyl groups [40,41]. This property allows them to efficiently “grasp” and neutralize Gtfs enzymes, inhibiting the synthesis of viscous glucans [42]. At the same time, it can adhere to the surface of the bacteria, altering its hydrophobicity and thus destabilizing the biofilm [43].

Tea is the most widely studied phenol-based CBP since it is commonly known as a functional beverage and has countless health benefits [44]. Traditionally, tea has been used in disease prevention for its mild anti-infective, anti-inflammatory, antioxidative, anti-aging, anticancer, and immunity-enhancing effects [45]. Through different processing procedures, tea is classified into white tea, green tea, oolong tea, and black tea. Green tea is an unfermented type produced by drying and steaming fresh tea leaves, in which the activity of oxidase is disrupted at high temperatures and therefore the integrity of tea polyphenols (TPs) is protected to the largest extent [46]. TPs refer to a mixture of various polyphenols, with catechin accounting for the largest proportion. It is believed that catechins, epigallocatechin (EGC) and epigallocatechin gallateare (EGCG) are responsible for many of its biological activities [47]. With regard to the anti-*S. mutans* effect, the majority of experiments reflected that tea exhibited inhibitory activity against *S. mutans*, whether in vitro or in vivo [48]. TPs mainly work by inhibiting microbial adhesion and activities of glucan synthesis through Gtfs [49], and fractions devoid of monomeric catechins additionally inhibit *S. mutans* through interacting with bacterial surface proteins [50]. To ensure tea’s physicochemical stability and bioavailability in the oral cavity, some researchers have even tried to develop tea-loaded chitosan nanoparticles, and they surprisingly observed a significant reduction in MIC and MBC against *S. mutans* [51].

Propolis is a resinous substance produced by bees from plant resins and their own secretions to repair hives, containing phenolic and flavonoid compounds, and has been used therapeutically for centuries [52]. It holds significant promise in dentistry due to its broad-spectrum antimicrobial and antibiofilm activity, attributed to its multifaceted mechanisms such as disrupting microbial cell membranes, inhibiting bacterial adhesion and enzyme function, and modulating host immunity [53,54]. Laboratory evidence confirms its efficacy against key oral pathogens like *S. mutans* [55,56]. However, clinical translation is hampered by variability in composition, extraction methods, and product formulations, as well as methodological limitations in existing studies [57]. Future efforts should prioritize extract standardization and rigorous clinical trials to establish its efficacy, safety, and optimal application protocols in dental practice [54,57].

Clove is a dried flower bud belonging to the Myrtaceae family, which has been traditionally used for food preservation and medicinal purposes [58]. In addition to serving as an ornamental plant or a spice, clove has been put into clinical use for years due to the anti-inflammatory [59] and antimicrobial effects [60]. The main extraction, clove essential oil (CEO), plays an important role in its pharmacological effect, of which eugenol is the major fraction, accounting for at least 50% [61]. The results of in vitro antibacterial tests suggested that CEO displayed potent antibacterial effect especially against *S. mutans*, manifested as a larger inhibition zone compared with other traditional herbs [62].

In folk medicine, with magnolol and honokiol as primary bioactive phenolic components, magnolia officinalis has a long history of use for the effects of promoting dampness, warming the body to relieve pain, and reducing adverse reactions and asthma [63]. It has been used in the treat of gastrointestinal disorders for hundreds of years, such as inflammation and ulcers [64]. In recent decades, magnolia officinalis has received great attention for its anti-*S. mutans* property. It was previously found that magnolol significantly suppressed Gtfs activity at a concentration as low as 0.5 mg/mL [65]. Additionally, Sakaue et al. [39] found that magnolol displayed favorable penetration ability and a strong bactericidal effect on biofilms of *S. mutans* with less cytotoxicity compared with chlorhexidine. It is also recommended by a few research studies to add a low concentration of *Magnolia officinalis* extract into chewing gum or mouthwash to enhance their ability to reduce oral pathogens in daily use [66].

### 2.4. Anthraquinone-Based CBPs

Anthraquinones are polycyclic compounds with an unsaturated diketone structure and have a variety of biological activities including anticancer, antibacterial, and antioxidant activities that reduce disease risk [67]. In CBPs with anthraquinone compounds as main bioactive ingredients, *Aloe vera* and *Polygonum cuspidatum* have drawn a great deal of interest. The planar conjugated aromatic structure of anthraquinones is the key to their anti-*S. mutans* function. This flat structural feature makes it easier to insert into the grooves of DNA double helices or transcriptional regulatory proteins, thereby interfering with the gene transcription program of *S. mutans* at the molecular level and inhibiting its cariogenicity [68].

*Aloe vera* ranks among the most widely used herbs due to its multiple cosmetic and medicinal properties [69]. Apart from application in skincare products, *Aloe vera* still has a series of benefits like anti-inflammatory, antidiabetic and antimicrobial effects [70]. Antibacterial tests showed that *S. mutans* was susceptible to *Aloe vera* among oral pathogens and it was proved by an in vitro model tested on extracted permanent molar that the application of *Aloe vera* gel could improve the enamel density and surface hardness [71]. In vivo studies also suggested herbal agents like *Aloe vera* could be employed as an oral antiseptic to reduce the occurrence of secondary caries [72].

*Polygonum cuspidatum*, the dried rhizome and root of a plant of the family *Polygonaceae*, has a long history as a medicinal plant [73]. It has traditionally been used to treat inflammation and hyperlipemia [74]. There are two main bioactive ingredients, emodin and physcion, which can reduce the acidogenic capacity of *S. mutans* through significantly inhibiting its glycolytic process [75].

### 2.5. Other Types

Other types refer to those anti-*S. mutans* CBPs with bioactive ingredients not belonging to any of the types mentioned above, such as mint and cinnamon. The bioactive ingredients are mostly volatilized; therefore, it is commonly suggested that their low molecular weight and hydrophobicity are the chemical basis for their function [76,77]. These small molecules may rapidly penetrate the lipophilic region of the bacterial cell membrane, physically disrupting the arrangement of the lipid bilayer and leading to cell membrane leakage and cell lysis [78].

Peppermint has been used in the food industry and oral health products like toothpastes, mouthwash, dental floss, etc., for years [79]. The chemical composition of peppermint is complex, but the main extractions of peppermint are peppermint essential oil (PEO) and non-volatile components [80]. PEO, primarily composed of menthol, exhibits a diverse pharmacological profile including anti-inflammatory, antibacterial, antiviral, immunomodulatory, antitumor, neuroprotective, antifatigue, and antioxidant activities [81]. Evaluation of the efficacy of PEO in caries prevention was also conducted, and it was claimed that mint was capable of inhibiting the growth and adherence of *S. mutans* and the activity of Gtfs [82,83,84,85].

Cinnamon is a well-known culinary spice that has also been traditionally applied in medical practices [86]. It has also shown potent antibacterial activity by directly damaging the cell membrane and reducing intracellular ATP [78]. The bioactive ingredients of cinnamon essential oil include cinnamaldehyde, eugenol, and linalool [87]. The potent antibacterial activity of cinnamon essential oil against *S. mutans* is primarily attributed to cinnamaldehyde, which damages bacterial membrane integrity, thereby increasing its permeability [88].

## 3. Mechanisms of Anti-Caries CBPs

The systematic classification of anti-caries CBPs based on their core bioactive components including organic acids, alkaloids, phenols, anthraquinones, and others provides a foundational framework for elucidating their mechanisms of action. The defining chemical features of each class, such as the ionizable carboxyl groups in organic acids, the complex nitrogenous structures in alkaloids, and the planar aromatic systems in phenols and anthraquinones, inherently govern their interactions with biological targets. These interactions enable CBPs to disrupt key virulence pathways of *S. mutans* in a targeted manner.

**Figure 2:**
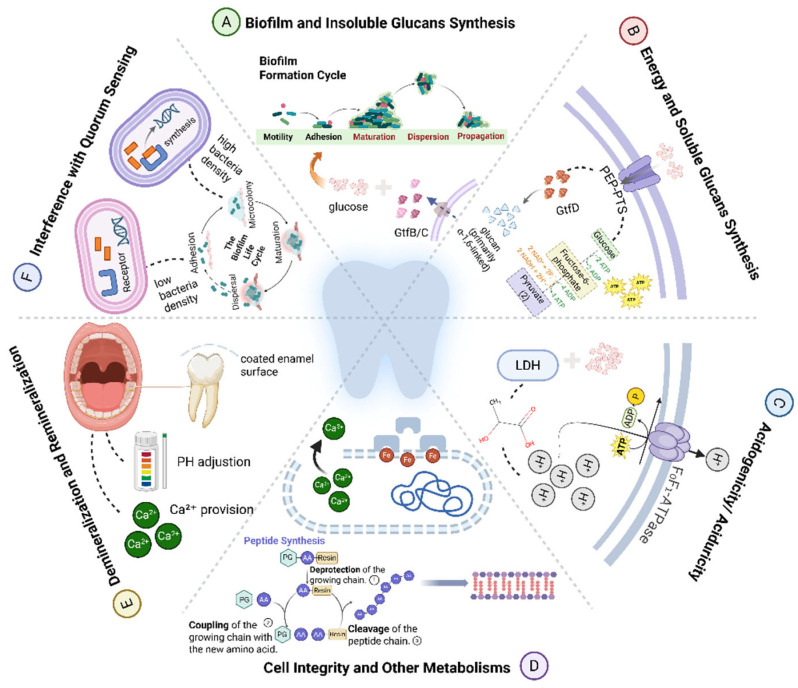
Schematic diagram of the anti-caries mechanisms of CBPs targeting *S. mutans.* Created in BioRender. Li, Y. (2026) https://BioRender.com/yzw7ukt (Accessed on 25 February 2026).

Specifically, the anti-caries efficacy of these compounds can be attributed to their concerted effects on several critical pathogenic processes: the synthesis of biofilm scaffolds and water-insoluble glucans, energy metabolism and soluble glucan production, acid generation and tolerance, bacterial cell integrity, intercellular communication via quorum sensing, and remineralization processes. The comprehensive regulatory network of these anti-caries mechanisms is illustrated in Figure 2. This mechanistic perspective underscores the advantage of CBPs as multi-targeted agents capable of modulating the cariogenic potential of the oral biofilm, offering a strategic approach to caries prevention that supports ecological balance rather than indiscriminate microbial elimination.

### 3.1. Biofilm and Insoluble Glucans Synthesis

The formation of dental plaque biofilm is a cornerstone of cariogenesis [89]. The process initiates with the adsorption of salivary proteins to enamel, forming an acquired pellicle [90]. *S. mutans*, as a primary pathogen, adheres to this pellicle via surface adhesins. Its key virulence factors, glucosyltransferases B and C (GtfB/C), then utilize sucrose to synthesize water-insoluble glucans (primarily α-1,3-linked) [91]. These glucans form a sticky, polymeric matrix that mediates firm microbial adhesion, facilitates coaggregation, and builds the structural scaffold of the biofilm, providing bacteria with enhanced resistance to antimicrobials and host defenses [92]. Disrupting this process by inhibiting enzyme activity, downregulating gene expression, or physically dismantling the biofilm can effectively undermines the foundation of dental caries. The specific effects of CBPs on biofilm scaffold and water-insoluble glucan synthesis are summarized in Table 2.

### 3.2. Energy and Soluble Glucans Synthesis

Beyond building the biofilm scaffold, *S. mutans* employs strategic carbohydrate metabolism for persistence [106]. The enzyme GtfD synthesizes water-soluble glucans (primarily α-1,6-linked) [107], which function not as adhesives but as extracellular energy reserves. These glucans can be catabolized during fasting periods, sustaining bacterial survival and acid production [108]. Concurrently, efficient glucose uptake via the phosphotransferase system (PEP-PTS) and its subsequent catabolism through glycolysis are paramount for rapid ATP generation and acidification [109]. By disrupting soluble glucan synthesis, blocking sugar transport, or inhibiting central metabolic pathways, CBPs can induce an energy crisis within the cariogenic community, weakening its competitivity and pathogenicity. The regulatory mechanisms of energy metabolism and soluble glucan production are detailed in Table 3.

### 3.3. Acidogenicity/Aciduricity

The direct demineralization of tooth enamel is importantly driven by bacterial acid production [114]. *S. mutans* ferments sugars to lactic acid predominantly via lactate dehydrogenase (LDH), defining its acidogenicity [115]. To thrive in this self-generated acidic niche, it employs the aciduricity mechanism, mainly through the F_0_F_1_-ATPase proton pump which exports intracellular protons to maintain pH homeostasis. The activity and assembly of this pump are tightly regulated, with genes like *atpD* encoding its critical subunits [116]. This dual capability to create and withstand acid is a masterstroke of cariogenic virulence. Inhibiting LDH activity or expression directly reduces the demineralizing agent, while impairing F_0_F_1_-ATPase function renders the bacterium vulnerable to acid stress, thereby restoring a more neutral oral ecology that is less conducive to caries progression. Molecular targets and effects related to acidogenicity and aciduricity are outlined in Table 4.

### 3.4. Cell Integrity and Other Metabolisms

The bacterial cell envelope, comprising the cytoplasmic membrane and cell wall, is essential for survival, governing selective permeability, structural integrity, and osmotic balance [122]. Disruption of this barrier leads to rapid cell death. CBPs employ diverse strategies to attack these cariogenic bacteria. Lipophilic compounds can integrate into and destabilize the lipid bilayer, causing rupture and ion leakage such as Ca^2+^ [123]. Others may act as chelators, sequestering essential trace metals like iron, and some may interfere with peptidoglycan synthesis, thereby weakening the cell wall. These direct, often broad-spectrum mechanisms target fundamental physiological processes, effectively eliminating pathogens regardless of their biofilm or planktonic state. Impacts on bacterial cell integrity and other metabolic pathways are presented in Table 5.

### 3.5. Demineralization Inhibition and Remineralization Promotion

Dental caries is fundamentally a dynamic process characterized by an imbalance between demineralization and remineralization [127]. The primary component of tooth enamel, hydroxyapatite (HA), dissolves when the local pH at the plaque–enamel interface falls below a critical level (pH ~5.5) due to bacterial acid production [114]. Conversely, remineralization occurs when calcium and phosphate ions from saliva or external sources precipitate back onto the enamel surface, especially under neutral or slightly alkaline conditions, repairing early subsurface lesions [128]. Unlike conventional anti-caries agents that primarily target bacteria, certain CBPs possess the unique ability to influence this mineral equilibrium directly. They can inhibit demineralization by protecting the enamel surface or collagen matrix, and more remarkably, enhance remineralization by acting as a source of mineral ions, facilitating their transportation to lesion sites, or templating the ordered growth of new hydroxyapatite crystals. This direct promotion of tooth tissue repair represents a pivotal and complementary strategy in the holistic prevention of dental caries. Strategies for interfering with quorum sensing systems are summarized in Table 6.

### 3.6. Interference with Quorum Sensing

Quorum sensing (QS) is a cell-density-dependent communication system that allows bacteria to coordinate group behaviors [133]. In *S. mutans*, the ComDE two-component system and the LuxS/AI-2 pathway are key QS systems regulating virulence traits such as biofilm development, acid tolerance, and bacteriocin production [134]. By interfering with QS through inhibiting signal synthesis, degrading autoinducers, or blocking receptor binding, CBPs can effectively disturb bacterial communication. Its anti-virulence strategy attenuating pathogenic behaviors without directly killing the bacteria reduces the selective pressure for resistance development and promotes a shift towards a healthier oral microbial equilibrium. The dual mechanism of demineralization inhibition and remineralization promotion is shown in Table 7.

## 4. Clinical Applications and Future Perspectives

Despite the promising in vitro and in vivo evidence, the direct clinical application of pure CBP extracts in dentistry remains limited. However, many kinds of CBPs have been incorporated into daily oral hygiene products and adjunctive therapies. Mouthwashes are one of the most mature clinical applications of CBPs. Propolis, tea polyphenols, and magnolia bark extracts are used in mouthwashes for their antibacterial and anti-inflammatory properties [66,143,144]. Dentifrices containing herbal compositions like peppermint, clove, and licorice are widely available commercially, offering a natural alternative for caries prevention and oral health [145,146,147]. Chewing gum with *Magnolia officinalis* extract can effectively reduce salivary *S. mutans* levels and plaque acidity [148,149]. Apart from that, there are related clinical research studies about CBPs as well, such as licorice and fennel seeds being added into chewing gums for preventing dental caries [18,150].

Despite the evidence mentioned above, to further promote the widespread use of CBPs in clinical treatment, it is necessary to address the unknown various chemical components and related toxicity issues associated with CBPs. The inherent variability in the chemical composition of CBPs, influenced by factors such as plant source, geographic origin, harvest time and processing methods, poses a significant barrier to ensuring consistent efficacy and safety across different batches. In addition, there is a lack of large-scale, double-blind randomized controlled trials (RCTs) to support the long-term efficacy and safe application of CBPs in caries prevention. To overcome these challenges and bring CBPs from bench to bedside, future RCTs are highly recommended. Furthermore, since synergistic combinations of CBPs with conventional agents (i.e., fluoride) could reduce the dose of both components and prevent side effects such as dental fluorosis, it is also promising to develop combined therapies and more efficient ways to deliver them precisely. Additionally, the combined effect often surpasses the effect of individual constituents. Deliberately exploring synergies between different CBPs, such as combining the enamel-protective organic acids of GC with biofilm-targeting phenolics from *Magnolia officinalis*, could enable the design of multi-targeted anti-caries formulations. Future research should employ systematic approaches like combination assays and network pharmacology to elucidate these interactions, optimize blending strategies, and develop evidence-based botanical therapies with enhanced clinical relevance.

In summary, this review provides a systematic and mechanistic framework for understanding anti-*S. mutans* CBPs by classifying them based on their primary bioactive components. This innovative approach moves beyond a simple listing of herbs and offers a clearer structure to elucidate their multi-targeted actions. Despite the unclarity of CBPs, in general, CBPs possess a significant anti-*S. mutans* effect and could benefit caries prevention in clinical practice.

## Figures and Tables

**Table 1:** Classification, active ingredients, chemical structure and source of anti-carious TCMs.

Classification	Name	Bioactive Ingredients	Chemical structure	Source
Organic acid-based	*Galla Chinensis*	Gallic acid, methyl gallate, polymeric polyphenols	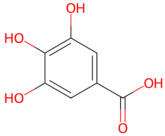 Gallic acid	The gall forming when the Chinese sumac aphid Baker (*Melaphis chinensis* Bell) parasitizes the leaves of *Rhus chinensis*
	*Radix glycyrrhizae* (licorice root)	Glycyrrhizic acid (Glycryrrhizin)	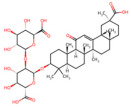 Glycyrrhizin	The dried roots and rhizomes of *Glycyrrhiza uralensis* Fisch, *Glycyrrhiza inflata* or *Glycyrrhiza glabra*
	*Lonicera japonica* (honeysuckle)	Chlorogenic acid, luteolin glycosides	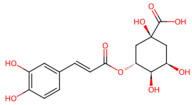 Chlorogenic acid	The dried flower buds or flowers in the early blooming stage of *Lonicera japonica*
	*Prunus mume* (plum)	Citric acid, mallic acid, chlorogenic acid	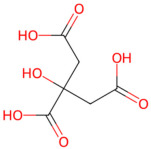 Citric acid	The dried nearly ripe fruit of *Prunus mume*
	*Anisum stellatum*	Shikimic acid	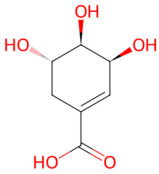 Shikimic acid	The dried ripe fruit of *Illicium verum*
Alkaloid-based	*Coptis chinensis*	Berberine	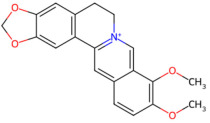 Berberine	The dried rhizome of *Coptis chinensis*
	*Chelidonium majus*	Chelerythrine	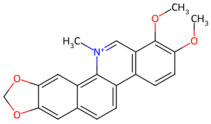 Chelerythrine	The whole herb of *Chelidonium majus*
	*Sophora flavescens*	Matrine, oxymatrine	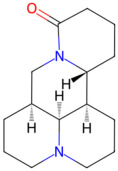 Matrine	The dried roots of *Sophora flavescens*
Phenol-based	Tea	Tea polyphenols (catechins, EGC, EGCG)	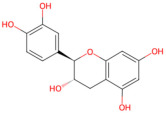 Catechin	The tender leaves or buds of the plant *Camellia sinensis*
	Propolis	Quercetin, rutin, apigenin	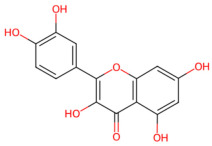 Quercetin	A natural product processed from the resinous substances secreted by honeybees
	Clove	Eugenol	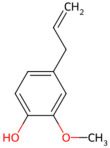 Eugenol	The dried flower buds of *Syzygium aromaticum*
	*Magnolia officinalis*	Magnolol, honokiol	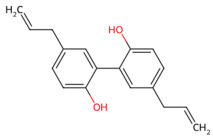 Magnolol 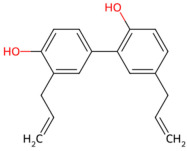 honokiol	The dried trunk bark, root bark or branch bark of *Magnolia officinalis*
Anthraquinone-based	*Aloe vera*	Aloe emodin, aloin	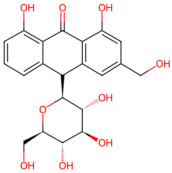 Aloin	The concentrated and dried juice of the leaves of *Aloe vera*
	*Polygonum cuspidatum*	Polydatin, emodin	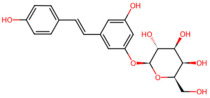 Polydatin	The dried rhizomes and roots of *Polygonum cuspidatum*
Other types	Peppermint	Menthol	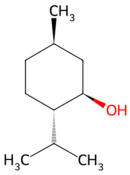 Menthol	The dried aerial parts of *Mentha haplocalyx*
	Cinnamon	Cinnamaldehyde, eugenol, linalool	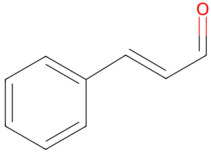 Cinnamaldehyde	The dried bark of *Cinnamomum cassia*
	Shaddock ped	Limonene, naringenin	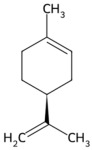 Limonene	The pericarp of *Citrus maxima*

Note: The chemical structures of bioactive ingredients are obtained from the website ChemSpider: Search and Share Chemistry—Homepage (https://www.chemspider.com/, Accessed on 25 February 2026).

**Table 2:** Effects of CBPs on biofilm formation and insoluble glucan synthesis.

Classification (Bioactive Component)	CBP (Representative Extract/Compound)	Effect on GtfB/C Activity	Effect on *gtfB/C* Expression	Effect on Biofilm Structure/Adhesion	References
I. Organic Acids					
1. Gallotannins/Phenolic acids	*Galla chinensis* (Aqueous extract, tannic acid)	↓ (Direct inhibition)	-	↓ (Complexes with pellicle, reduces affinity)	[13,93]
2. Triterpenoid saponins	*Radix Glycyrrhizae* (Glycyrrhizic acid)	↓	-	↓ (Surface coating effect)	[94,95,96]
3. Chlorogenic acid	*Lonicera japonica* (Chlorogenic acid)	↓ (Via QS inhibition)	-	-	[20]
4. Shikimic acid	*Anisum stellatum* (Shikimic acid)	-	↓	↓ (Damages cell membrane)	[25]
II. Alkaloids					
1. Isoquinoline alkaloids	*Coptis chinensis* (Berberine chloride)	-	-	↓ (Downregulates *srtA*, *gbpC*; inhibits metabolic activity)	[28]
2. Isoquinoline alkaloids	*Chelidonium majus* (Chelerythrine)	-	-	↓ (Reduces adhesion ability)	[34,97]
III. Phenols					
1. Tea polyphenols	Tea (EGCG, catechins)	↓ (Non-competitive, binds glucan domain)	↓	↓ (Alters hydrophobicity & aggregation)	[42,98,99,100]
2. Flavonoids/Phenolics	Propolis (PEOME, apigenin)	↓	-	↓ (Increases hydrophobicity, damages membrane)	[56,74]
3. Phenolic lignans	*Magnolia officinalis* (Magnolol)	↓ (Non-competitive, binds glucan domain)	-	↓ (Penetrates biofilm)	[39,65]
4. Eugenol derivatives	Clove (Clove essential oil)	-	-	↓ (Damages cell membrane)	[61,101]
IV. Anthraquinones					
1. Anthraquinone glycosides	*Aloe vera* (Gel)	-	-	↓ (Inhibits growth and adherence)	[102]
2. Emodin, Physcion	*Polygonum cuspidatum* (Emodin)	-	-	-	[103] -
V. Others					
1. Cinnamaldehyde, etc.	Cinnamon (Cinnamaldehyde)	-	↓	↓ (Alters hydrophobicity & aggregation)	[104,105]

**Table 3:** Effects of CBPs on energy metabolism and soluble glucan synthesis.

Classification (Bioactive Component)	CBP (Representative Extract/ Compound)	Effect on GtfD/ Soluble Glucan	Effect on Sugar Uptake (PEP-PTS)	Effect on Glycolysis/Energy Metabolism	References
I. Organic acids					
1. Gallotannins/ Phenolic acids	*Galla chinensis* (Gallic acid)	↓ (Downregulates *gtfD*)	-	-	[110]
2. Triterpenoid saponins	*Radix Glycyrrhizae* (Glycyrrhizic acid)	↓ (Inhibit Gtfs)	-	-	[111]
II. Alkaloids					
1. Isoquinoline alkaloids	*Coptis chinensis* (Berberine)	-	-	↓ (Inhibits biofilm metabolic activity)	[28]
III. Phenols					
1. Tea polyphenols	Tea (Catechins, TPs)	-	↓ (Blocks EIIC transporter)	↓ (Downregulates glycolysis & TCA cycle)	[43,112]
IV. Anthraquinones					
1. Emodin, Physcion	*Polygonum cuspidatum* (Bioassay-guided fraction)	-	-	↓ (Produces anti-acidogenic substances, inhibits glycolytic process)	[75,113]

**Table 4:** Effects of CBPs on acidogenicity and aciduricity of *S. mutans*.

Classification (Bioactive Component)	CBP (Representative Extract/Compound)	Effect on LDH (Acidogenicity)	Effect on F_0_F_1_-ATPase (Aciduricity)	References
I. Organic acids	*Galla chinensis* (Extract)	- (Limits acid accumulation)	-	[12]
II. Alkaloids				
1. Isoquinoline alkaloids	*Coptis chinensis* (Berberine hydrate)	↓ (Downregulates *ldh* expression)	-	[27]
III. Phenols				
1. Tea polyphenols	Tea (Catechins, EGCG)	↓ (Inhibits activity; blocks substrate)	↓ (Inhibits activity & *atpD* expression)	[112,117,118]
2. Phenolic lignans	*Magnolia officinalis* (Honokiol)	↓ (Downregulates *ldh* expression)	-	[119]
3. Flavonoids/Phenolics	Propolis (Essential oil, PEOME, ethanol extract)	↓ (Downregulates *ldh*; inactivates leaked enzyme)	↓ (Inhibits activity, disrupts pH gradient)	[55,56,120]
IV. Anthraquinones				
1. Anthraquinone glycosides	*Aloe vera* (Gel)	-	-	[102]
V. Others				
Cinnamaldehyde, trans-Cinnamaldehyde	Cinnamon	↓ (Inhibits glycolytic enzymes)	↓ (Suppresses *atpD* expression)	[105,121]

**Table 5:** Effects of CBPs on bacterial cell integrity and metabolism.

Classification (Bioactive Component)	CBP (Representative Extract/Compound)	Effect on Cell Membrane	Effect on Ion Homeostasis/Metals	Effect on Cell Wall/Other Metabolism	References
I. Organic acids					
1. Gallotannins/ Phenolic acids	*Galla chinensis* (Gallic acid)	↓ (Disrupts bilayer, causes Ca^2+^ efflux)	↓ (Iron chelation by tannic acid)	-	[6,104,124]
2. Shikimic acid	*Anisum stellatum* (Shikimic acid)	↓ (Alters membrane proteins)	-	-	[25]
III. Phenols					
1. Tea polyphenols	Tea (EGCG, TPs)	↓ (Reduces hydrophobicity, impairs permeability)	-	↓ (Disrupts peptidoglycan cross-linking)	[43,124]
2. Flavonoids/Phenolics	Propolis (Extract)	↓ (Alters hydrophobicity, forms pores)	-	-	[125]
3. Eugenol derivatives	Clove (Clove essential oil)	↓ (Penetrates and damages lipids)	-	-	[60,126]
V. Others					
1.Cinnamaldehyde, etc.	Cinnamon (Essential oil)	↓ (Damages membrane, reduces ATP)	-	-	[78]

**Table 6:** Effects of CBPs on quorum sensing systems in *S. mutans*.

Classification (Bioactive Component)	CBP (Representative Extract/Compound)	Effect on ComDE System	Effect on LuxS/AI-2 System	Effect on Other QS-Related Elements	References
I. Organic acids					
1. Gallotannins/ Phenolic acids	*Galla chinensis* (Polyphenols)	-	-	↓ (Interacts with QS signals, undermines competence)	[129]
2. Chlorogenic acid	*Lonicera japonica* (Chlorogenic acid)	-	↓ (Blocks AI-2 sensing)	↓ (Downregulates *vicK*)	[20]
II. Alkaloids					
1. Isoquinoline alkaloids	*Coptis chinensis* (Berberine)	↓ (Suppresses *comX* expression)	-	-	[28]
III. Phenols					
1. Tea polyphenols	Tea (EGCG)	-	↓ (Downregulates *luxS* expression)	-	[130]
2. Flavonoids/Phenolics	Propolis (CAPE)	-	-	↓ (Downregulates *vicK*, *vicR*, *ccpA*)	[131,132]
V. Others					
1. Cinnamaldehyde, etc.	Cinnamon (trans-Cinnamaldehyde)	↓ (Downregulates *comDE*)	↓ (Downregulates *luxS*)	-	[105]

**Table 7:** Effects of CBPs on demineralization and remineralization processes.

Classification (Bioactive Component)	CBP (Representative Extract/Compound)	Effect on Demineralization	Effect on Remineralization	Proposed Mechanism	References
**I. Organic acids**					
1. Gallotannins/Phenolic acids	*Galla chinensis* (GC extract, Gallic Acid, Tannic acid)	↓ (Inhibits ion diffusion)	↑↑ (Significant enhancement)	1. Provides Ca^2+^ ions. 2. Forms “enamel organic matrix–GC–Ca^2+^” complex to transport ions. 3. Forms “GC–dentin matrix” complex to stabilize collagen.	[7,135,136,137,138]
2. Triterpenoid saponins	*Radix Glycyrrhizae* (Glycyrrhizic acid)	↓ (Reduces enamel dissolution)	-	Surface coating effect that limits acid access.	[139]
**III. Phenols**					
1. Flavonoids/Phenolics	Propolis (Extract)	-	↑ (Potential effect suggested)	May aid in mineral deposition.	[140,141]
2. Eugenol derivatives	Clove (Clove Essential Oil)	-	↑ (Potential effect suggested)	May operate on de/remineralization balance.	[60,142]
**IV. Anthraquinones**					
1. Anthraquinone glycosides	*Aloe vera* (Gel)	-	↑ (Improves enamel density/hardness)	Application of gel improves surface microhardness in vitro.	[71,141]

## Data Availability

No new data were created or analyzed in this study.

## Abbreviations

**Abbreviations**	**Full name**
CBP	Chinese botanical product
EPS	Extracellular polysaccharides
GC	*Galla Chinensis*
GA	Gallic acid
MG	Methyl gallate
Gtfs	Glucosyltransferases
GtfB/C	Glucosyltransferase B/C
GtfD	Glucosyltransferase D
QS	Quorum sensing
MIC	Minimal inhibitory concentration
MBC	Minimal bactericidal concentration
TP/TPs	Tea polyphenols
EGC	Epigallocatechin
EGCG	Epigallocatechin gallate
PEOME	Propolis essential oil microemulsion
CAPE	Caffeic acid phenethyl ester
CEO	Clove essential oil
PEO	Peppermint essential oil
SEM	Scanning electron microscopy
TEM	Transmission electron microscopy
PEP-PTS	Phosphoenolpyruvate–carbohydrate phosphotransferase system
TCA	Tricarboxylic acid
GTase	Guanylyl transferase
Ftf	Fructosyltransferase
Gbp	Glucan binding protein
OTE	Oolong tea extract
OTF10	Oolong tea polyphenol 10
AI-2	Autoinducer-2
LDH	Lactate dehydrogenase
AFM	Atomic force microscope
XRD	X-ray diffraction
SMH	Surface microhardness
%SMHR	Surface microhardness recovery
CHX	Chlorhexidine
HA	Hydroxyapatite
RCTs	Randomized controlled trials
